# The Balloon Analog Insurance Task (BAIT): A Behavioral Measure of Protective Risk Management

**DOI:** 10.1371/journal.pone.0021448

**Published:** 2011-06-28

**Authors:** Brian G. Essex, Carl W. Lejuez, Rebecca Y. Qian, Katherine Bernstein, David H. Zald

**Affiliations:** 1 Department of Psychology, Vanderbilt University, Nashville, Tennessee, United States of America; 2 Department of Psychology, University of Maryland, College Park, Maryland, United States of America; 3 Department of Psychiatry, Vanderbilt University, Nashville, Tennessee, United States of America; University of Minnesota, United States of America

## Abstract

Prior methods used to assess individual differences related to risk have not focused on an important component of risk management: how willing individuals are to pay for or take actions to insure what they already have. It is not clear whether this type of protective risk management taps into the same individual differences as does risk taking propensity measured by existing risk taking tasks. We developed a novel task to assess protective risk management, the Balloon Analog Insurance Task (BAIT), which is modeled after the Balloon Analog Risk Task (BART). In the BAIT, individuals are forced to decide how much money they are willing to pay in order to insure a specific fraction of their prior winnings given changing but imprecise levels of risk of monetary loss. Participants completed the BART and BAIT for real monetary rewards, and completed six self report questionnaires. The amount of insurance purchased on the BAIT was positively correlated with scores on the Intolerance of Uncertainty Scale and on the Checking scale of the revised Obsessive Compulsive Inventory. Conversely, the amount of insurance purchased was negatively correlated with scores on the Domain Specific Risk Taking Questionnaire, and on the Psychopathic Personality Inventory (PPI). Furthermore, relationships between insurance purchased and these scales remained significant after controlling for the BART in linear regression analyses, and the BART was only a significant predictor for measures on one scale - the PPI. Our results reveal that behavior on the BAIT taps into a number of individual differences that are not related to behavior on another measure of risk taking. We propose that the BAIT may provide a useful complement to the BART in the assessment of risk management style.

## Introduction

Managing risk is a critical part of human endeavors. Decisions requiring an assessment of risk range widely from whether to wear a seat belt or have unprotected sex to how to invest ones money. Such decisions can have widespread economic and public health consequences, impacting both the global economy and the spread of disease.

Attempts to measure individual differences in risk taking have often relied on self-report measures. However, recently objective behavioral measures have gained prominence due to their ability to directly assess risk-taking behavior, at least as regards to monetary risk. The most widely used of these measures ask participants to make choices between options that could lead to making more money, but also risk losing money, relative to a safer option. For instance in the Balloon Analogue Risk Task (BART) [1], individuals have to decide whether to fill balloons up further to earn more money, but risk losing all of the money for the trial if the balloon explodes. Similarly, in the Iowa Gambling Task [2] participants have to choose between card decks, some of which have larger wins, but also larger losses relative to safer decks which have lower gains but substantially lower risks of losses. Both of these tasks have been shown to have significant correlates including psychopathological traits or symptoms as well as the likelihood of participating in illegal or dangerous activities [1], [3], [4], [5]. A number of additional tasks have been developed for neuroeconomic research, often characterized by decision making under situations in which risk (probability of gains vs. losses) is known [6], [7], [8], [9]. Most of these tasks are formulated such that they measure an individual's readiness to risk a loss in order to receive a potential gain.

However, there is another major element of risk management that has received little attention in individual differences research. Specifically, how much are individuals willing to pay for or take actions in order to insure or protect their current possessions. Such protective risk management plays a major role in the economy, as is attested by the size of the insurance industry. Parallels are seen in taking care of one's health or protecting against pregnancy, in that the individual is willing to take action, or pay a price proactively in order to avoid the risk of potentially larger negative consequences in the future. The extent to which this type of protective risk management taps the same individual differences as those seen in tasks such as the Iowa Gambling Task or BART is not known. However, there are reasons to think that tendencies to engage in protective risk management may capture different correlates than tendencies to engage in risk taking, given that humans often demonstrate different behavioral biases for situations involving losses and gains [10], [11]. For instance, in low probability loss situations, people frequently behave in risk averse ways (accepting certain “insurance" costs to avoid larger losses even though the insurance costs are overpriced given the probability of loss), while in similarly low probability gain situations they perform in risk seeking ways (buying lottery tickets even though the price of the ticket costs more than the expected value of the lottery).

Here we describe a behavioral measure of protective risk management. The new task, which we have dubbed the Balloon Analogue Insurance Task, or BAIT for short, is modeled on the BART. During the BAIT, individuals are repeatedly forced to decide how much they are willing to pay in order to protect a specific fraction of their prior monetary winnings given changing, but imprecise levels of risk of loss. We propose that this task may provide a useful compliment to the BART, or other risk assessment tasks by assessing a unique aspect of risk tolerance. Additionally we provide data supporting that it captures distinct variance from the BART and provide preliminary data that it outperforms the BART in predicting individual differences in attitudes towards uncertainty, attitudes towards engaging in risky behaviors, and obsessive compulsive symptoms.

## Materials and Methods

### Participants

131 individuals (53.44% female) between the ages of 18 and 30 (*M* Age = 20.19 years, *SD* = 1.99) from Vanderbilt University and the Nashville community participated in this study. All of the participants reported having no history of neurological or psychiatric disorders or head trauma. Complete data were not available for 3 subjects who were excluded from further analyses.

### Ethics Statement

This study was approved by the Institutional Review Board of Vanderbilt University, and all participants completed approved written informed consent.

### Balloon Analogue Risk Task

Immediately after consent, participants completed the BART, following procedures described in Lejuez et al. [1]. On each trial, participants saw a balloon on the computer screen and could click the mouse on a box under the balloon to pump up the balloon. Each mouse-click increased the size of the balloon and added one cent to a temporary bank. Participants could decide to cash out after each mouse click by clicking on a box labeled “collect $$$" in which case all money accrued from pumping up the current balloon was transferred to a permanent bank. However, each balloon had an explosion point between 9 and 121 pumps (*M* = 64 pumps) that was unknown to the participants. Participants were told some balloons might pop after just one pump, but others might not pop until the balloon filled the entire screen. If the balloon was pumped up to its explosion point, the balloon exploded, with a “pop" sound effect and participants lost all money from the current trial. After the balloon popped or the participant cashed out, the next trial began. If the participant cashed out, the money from the trial was added to the total amount of money listed in a box labeled “Total Bank". There were 30 such trials in this task, which took approximately 10–20 minutes to complete. Each individual trial took approximately 10–45 seconds.

In order to investigate performance on the BART, we examined performance on two variables measuring risk propensity on the task: total number of balloon explosions (BART_Explo) and adjusted average number of pumps (BART_PAdjAvg), which is the average number of pumps on trials in which the balloon did not explode. The number of explosions on the BART has been considered to be a measure of more maladaptive risk taking than the adjusted number of pumps, because while the number of explosions indicates trials in which risks taken exceeded a beneficial level, the adjusted number of pumps indicates risk taking that was rewarded [4]. Since individuals began the BAIT with their earnings from the BART, we also examined how much individuals earned on the BART in dollars (BART_$Total) in order to see if the amount earned on the BART influenced the amount of insurance purchased on the BAIT.

### Balloon Analogue Insurance Task

After finishing the BART, participants completed the BAIT. Each participant's total earnings from the BART were carried over to the BAIT, such that they began the BAIT with their total BART winnings in their permanent bank. This was done because we wanted subjects to have a sense of ownership for this money. Before beginning the BAIT, participants were informed that they would again be pumping up balloons and were given additional information regarding the structure of balloon pops. As on the BART, participants were instructed that each balloon could pop between 1 and 128 pumps. However, unlike on the BART, for the BAIT participants were told that the average number of pumps before a balloon explodes is 64. Critically, unlike the BART, during the BAIT participants did not get to choose the number of pumps per balloon; rather, before each trial, participants were instructed how many times they would have to pump the specific balloon. As on the BART, if a balloon popped, participants lost all the money they had accrued for that trial. Participants were also told that during the course of the task there was one “unlucky" balloon, and that if the unlucky balloon popped they would lose all their earnings on that trial and all previous earnings (i.e. all of the earnings in their permanent bank). They were further instructed that if the unlucky balloon occurred on a trial in which there was no explosion, they would not know whether or not the unlucky balloon had been presented.

After seeing the number of required pumps for a given trial on the BAIT, participants were given the option to purchase insurance to protect against loss of money in their permanent bank in the event that the unlucky balloon popped on that trial. This insurance did not offer any protection against losing potential earnings from their temporary bank if the balloon popped on a given trial as it only covered money in the permanent bank. Participants could choose to insure 0, 30, 60, 90, or 100% of the total earnings in their permanent bank. Depending on the amount of insurance they chose, they were immediately deducted 0, 20, 30, 40, or 50 cents respectively, which was taken out of their permanent bank. Participants were told that if they purchased the maximum level of insurance on every trial, they would spend a substantial portion of their earnings. Throughout the trial, the total amount of insurance purchased for that trial was listed in a box labeled “Insurance Level".

**Figure 1:**
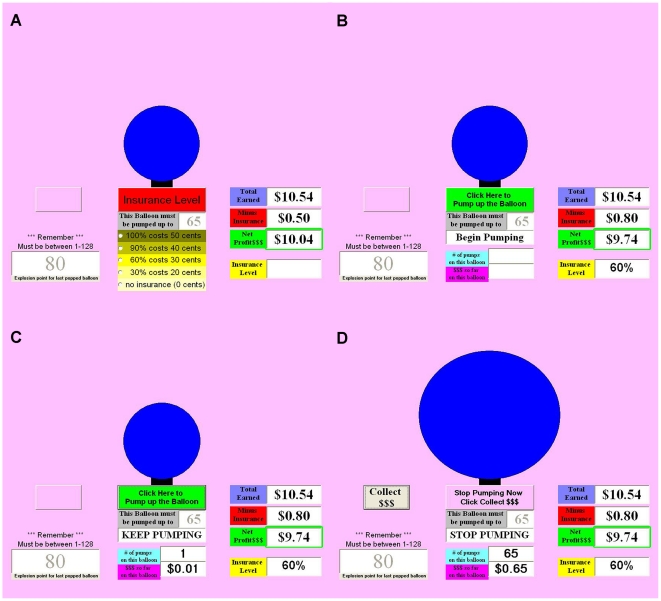
Task structure for one trial of the BAIT. A) At the beginning of each trial, the participant indicates how much insurance they would like to purchase for that trial. Additionally, they see how many pumps will be required for the current trial (here it is 65), and the number of pumps for the last popped balloon (here it was 80). B) After purchasing insurance, the participant is instructed to begin pumping up the balloon. C) The participant continues to pump up the balloon until the required number of pumps has been reached. D) After the required number of pumps has been reached, the balloon either explodes or the participant is instructed to click on the “Collect $$$" box to collect the money acquired from that trial. In this example, the balloon did not pop.

Figure 1 provides a schematic of the trial structure of the BAIT. After being given the opportunity to purchase insurance, the trial began. As with the BART, each mouse-click increased the size of the balloon and added one cent into that trial's temporary bank. Once the prescribed number of clicks had been reached, the balloon either exploded in which case participants lost the money for that trial or they were told to click “Collect $$$", in which case the money from that trial was transferred to their permanent bank. The balloon never popped before the prescribed number of clicks had been reached (i.e. if it popped it always popped after the last prescribed pump).

The task contained 20 trials and participants were told this before beginning. Each trial took approximately 10–45 seconds, and the entire task took approximately 10–20 minutes to complete. Each participant received the same balloons in this task in the same predetermined order, and the number of pumps required for each of the 20 balloons was the same for all participants and ranged from 18 to 118. Additionally, the explosion points for the balloons were the same across participants, and 10 of the balloons always popped. The distribution of explosions was such that the number of pumps required was not fully informative of risk, as a greater number of balloons popped below than above the mean number of required pumps (i.e. 6 and 4, respectively).

Although participants were told that one of the balloons on the task was unlucky, for the version of the BAIT used here, there was in fact no unlucky balloon (we note that this deception was approved by the Vanderbilt University Institutional Review Board). Before and during each trial on the BAIT, subjects also saw a box labeled “Explosion Point for last Popped Balloon", which listed the number of pumps on which the last popped balloon exploded. Additionally, participants always saw a box listing the total amount of money earned throughout the task, a box listing the amount spent on insurance, and the amount in their permanent bank (“Net Profit $$$") which was equal to total earnings minus insurance payments. After completing the BAIT, participants were able to keep all of the money accrued in their permanent bank which was paid to them at the end of the session. At the end of the entire session, participants were informed that there had in fact been no unlucky balloon.

To measure BAIT performance, we examined three variables. The chief dependent variable was the total amount of insurance purchased on all trials (BAIT_Ins). We additionally divided trials based on whether they were above or below the mean explosion point, categorizing the 11 trials with pumps of less than 64 as low risk (LR) trials, and the 9 trials with 65 or more pumps as high risk (HR) trials in order to see if there were any differential associations based on the level of perceived risk. Thus, in addition to BAIT_Ins, we analyzed the sum of insurance purchased on LR trials (BAIT_LR) and on HR trials (BAIT_HR). As a more precise measure of the relationship between the riskiness of the trial and amount of insurance purchased, we calculated the correlation between the amount of insurance purchased and the number of pumps required on each trial. We also calculated the correlation between the amount of insurance purchased and the trial number, which could also reveal a relationship between trial risk and insurance. Given that the amount of money that could be lost if the unlucky balloon pops is larger after more money is accrued, and more money is accrued over time, the negative consequence of the unlucky balloon popping is greater as the task progresses. Additionally, subjects may believe that the probability of an unlucky balloon occurring increases because they have yet to receive one. We note however, that a relationship between trial number and insurance purchased could also reflect changes in familiarity with the task.

As a measure of internal consistency on the BAIT, we computed the correlation between the amount of insurance purchased on odd and on even trials. Since odd and even trials required a similar number of pumps, a positive correlation between the amounts of insurance purchased on these two sets of trials would be indicative of consistent responding.

### Self Report Measures

Immediately after completing the BAIT, individuals completed six self-report questionnaires. We administered the Discomfort Intolerance Scale (DIS) [12], the Penn State Worry Questionnaire (PSWQ) [13], and the short version of the Obsessive-Compulsive Inventory (OCI-R) [14] to assess the level of anxious symptoms in individuals. The DIS is a measure of discomfort intolerance for which higher total scores indicate greater intolerance. On the DIS, individuals rate on a seven point scale how much physical discomfort they can tolerate and how avoidant they are of such discomfort. The PSWQ assesses trait levels of worry with higher scores indicating greater tendencies to worry. On the PSWQ, individuals rate how typical or characteristic various statements about worry are for them. Items are rated on a 5 point scale, from not at all to very typical. The OCI-R contains 18 items that assess for symptoms of obsessive compulsive disorder by asking individuals how distressed or bothered they have been in the past month by various experiences. The items are rated on a five point scale, from not at all distressed to extremely distressed and fall across six domains: Checking, Hoarding, Neutralizing, Obsessing, Ordering, and Washing. Higher scores reflect greater syptomatology. We computed total scores in each domain, and total overall scores.

We administered the English language version of the Intolerance of Uncertainty Scale (IUS) to assess how uncomfortable individuals were with uncertainty [15], [16]. On the IUS, individuals rate how characteristic each item is of them on a five point scale, from not at all characteristic to entirely characteristic. Higher total scores on the IUS reflect greater intolerance of uncertainty.

To measure the extent that participants engage in risky behavior, we administered the Domain Specific Risk Taking Questionnaire (DOSPERT) [17]. This questionnaire contains 50 items for which individuals indicate the likelihood that they would engage in various risky behaviors in five commonly encountered domains: Ethical, Financial, Health/Safety, Recreational, and Social. Items are rated on a 5 point scale from extremely unlikely to extremely likely, with higher scores indicating a greater likelihood of taking risks in that domain. In addition to looking at scores within each domain, we examined the total score on the DOSPERT, which provides a nonspecific index of the likelihood of risk taking.

We administered the short version of the Psychopathic Personality Inventory (PPI), a 56 item questionnaire that assesses psychopathic personality traits across eight subscales [18]. Although the PPI has not been used widely in research on risk, it captures several personality traits associated with psychopathy that may be particularly relevant to individual differences in risk management. Of note, psychopathy is characterized by a lack of planfullness and appreciation of risk that appears close to the construct we intended to capture with the BAIT. On the PPI, individuals rate on a four-point scale how true a number of statements are for them. We looked at three scores on the PPI: the total score and the total score on two factors. The separation of the PPI into two factors is based on work by Wilson and colleagues [18] finding that the Social Potency, Coldheartedness, Fearlessness, Impulsive Noncomformity, and Stress Immunity scales load onto one factor (PPI Factor 1) while the Machievellian Egocentricity, Blame Externalisation, and Carefree Nonplanfulness load onto a second factor (PPI Factor 2). We created these factors by adding the z-transformed scores for the scales associated with each factor. Higher scores on the total score and on each factor score reflect greater levels of psychopathic personality traits. The PPI was added to the study after approximately 1/4 of subjects had been run, and thus analyses of this scale are based on 95 participants rather than the complete sample.

### Missing Data

In a small number of cases, individuals failed to complete one or more items on various questionnaires. To construct total scores on the self-report questionnaires for individuals with missing data, we replaced the missing values with the average values for that participant on that questionnaire. With the exception of the IUS, all scales had complete data from more than 95% of participants. As discussed in results, restriction of the IUS analysis to just those participants who answered all questions did not significantly alter the results. Across analyses, all scale scores that were outliers (>3 standard deviation from mean score) were removed from analyses.

## Results

### Performance on the BAIT

For means and standard deviations of performance variables on the BART and BAIT, see Table 1. Participants purchased more insurance on the high risk trials than on the low risk trials (t(127) = 6.60, p<.001). Across trials, there was a positive correlation between the amount of insurance purchased and the required number of pumps (r = .32, p<.001) and between the amount of insurance purchased and the number of the trial (r = .21, p<.001). The amount of insurance purchased on the odd and even trials of the BAIT was strongly correlated (r = .89, p<.001), providing evidence for high internal consistency.

**Table 1:** Correlations between the BART and BAIT for all Participants.

Variable	Mean	SD	BAIT_Ins	BAIT_LR^nn^	BAIT_HR
BAIT_Ins	4.28	1.94	-	.78+	.90+
BAIT_InsLR^nn^	1.87	1.18	.78+	-	.51+
BAIT_InsHR	2.37	.93	.90+	.51+	-
BART_PAdjAvg	39.59	15.09	−.10	−.06	−.04
BART_Explo^nn^	10.35	4.01	−.06	−.06	−.01
BART_$Total^nn^	7.22	1.75	−.03	−.03	.01

BAIT_Ins: Total amount of insurance purchased in dollars on the BAIT. BAIT_InsLR, BAIT_InsHR: Amount of insurance purchased in dollars on the BAIT on low risk trials and high risk trials, respectively. BART_PAdjAvg: Adjusted Average number of pumps per balloon on the BART. BART_Explo: Total number of exploded balloons on the BART. BART_$Total: Total dollars earned on the BART. nn: variable is significantly non-normal according to Kolmogorov-Smirnov test, and all correlations with this variable are values of Kendall's Tau. All other correlations are Pearson correlations. Number of participants (N) is 128 for all correlations. + p<.001 (2-tailed).

We investigated the relationship between measures on the BART and BAIT and found that there were no significant correlations between measures on the two tasks, suggesting that the two tasks tap into different underlying constructs. Additionally, the lack of correlations between the amount of money earned on the BART and performance measures on the BAIT reveals that the amount of money participants had at the beginning of the BAIT did not influence their performance. In contrast to the lack of correlations between tasks, all three measures on the BAIT had positive correlations with all of the other measures on the BAIT and these correlations were significant at a level of p<.001. For a list of correlations between the BART and BAIT, see Table 1. None of the BAIT variables were significantly correlated with gender or age.

To determine which individual differences were related to the purchase of insurance on the BAIT, we examined correlations between the self-report scores listed in the methods section with BAIT_Ins, BAIT_InsLR, and BAIT_InsHR. This only revealed one significant relationship. Higher scores on the OCI-R Checking scale were associated with greater purchase of insurance on the high risk trials (Kendall's Tau = .13, p<.05).

### Relationships with BAIT for those responsive to risk

Although for the majority of individuals (110 out of 128), there was a positive correlation between amount of insurance purchased on trials of the BAIT and the riskiness of the trial as defined by the required number of pumps, 18 individuals showed either no relationship or a negative relationship between insurance purchased and riskiness. These 18 individuals did not appear to be responsive to the key predictor of risk on the BAIT, which could either reflect a lack of engagement with the task or a misunderstanding of how risk varied with the number of required pumps. Since the inclusion of these participants in the initial correlation analyses may have obscured relationships between the purchase of insurance on the BAIT and other variables, we reran the correlation analyses with the BAIT by only including the 110 individuals who had positive correlations between required number of pumps and insurance purchased per trial.

This time insurance purchased on the BAIT was significantly correlated with a number of individual difference measures. Insurance purchased overall on the BAIT was negatively correlated with the total score on the PPI (r = −.23, p<.05), the score on PPI Factor 1 (r = −.28, p<.05), the total score on the DOSPERT (r = −.19, p<.05), and the score on the DOSPERT Health/Safety scale (r = −.21, p<.05). The amount of insurance purchased on the low risk trials of the BAIT was positively correlated with the total score on the IUS (Kendall's Tau = .14, p<.05), and negatively correlated with the total score on the PPI (Kendall's Tau = −.17, p<.05) and the score on the DOSPERT Health/Safety scale (Kendall's Tau = −.14, p<.05). The correlation between the OCI-R checking scale and the insurance purchased on the BAIT high risk trials remained significant (Kendall's Tau = .14, p<.05); and the amount of insurance purchased on these high risk trials was negatively correlated with the PPI Factor 1 Score (r = −.27, p<.05). For a full list of correlations see Table S1. As before, none of the measures of insurance purchased on the BAIT were correlated with age or gender.

Next, we performed linear regression analyses with the forward regression method in PASW Statistics 18 (SPSS Inc., Chicago, IL) on self-report scores significantly correlated with the BAIT to test whether variables on the BAIT were predictive of individual differences in the subset of participants who had a positive correlation between the amount of insurance purchased and number of required pumps on BAIT trials. For all regressions, BAIT_Ins, BAIT_InsLR, and BAIT_InsHR were entered as independent variables. Criteria for entry of an independent variable into the regression was taken as probability of F less than.05 and criteria for removal was probability of F greater than.10. We additionally entered the adjusted average number of pumps on the BART (BART_PAdjAvg), the total number of explosions on the BART (BART_Explo), and the amount of money earned on the BART (BART_$Total) as predictors in all regressions in order to examine whether the BAIT variables had predictive validity beyond that afforded by the BART.

With the exception of the total score on the PPI, all scales that had been significantly correlated with the BAIT were significantly predicted by one of the three BAIT variables in the regressions and these predictions were in the same direction as were the correlations. Greater insurance purchased on the BAIT predicted lower total scores on the DOSPERT (β = −.19, p<.05), lower total scores on the DOSPERT Health/Safety scale (β = −.21, p<.05), and lower scores on PPI Factor 1 (β = −.25, p<.05). Additionally, greater insurance purchased on the BAIT high risk trials predicted higher levels of symptoms on the OCI-R checking scale (β = .21, p<.05), while greater insurance purchased on the BAIT low risk trials predicted higher scores on the IUS (β = .20, p<. 05). Two scores were predicted by the number of explosions on the BART, while none were predicted by the adjusted average number of pumps on the BART or the amount of money earned on the BART. Greater number of explosions on the BART predicted both greater PPI total scores (β = .30, p<.01) and greater scores for PPI Factor 1 (β = .23, p<.05). For a list of significant regressions see Table 2.

**Table 2:** Regressions for which BART or BAIT significantly predicted risky behaviors.

Predicted Variable	Predictor Variables	B	SE B in Model	β
DOS_Total	Constant+	2.72	.10	
	BAIT_Ins*	−.04	.02	−.19
DOS_Health/Safety	Constant+	2.55	.15	
	BAIT_Ins*	−.07	.03	−.21
IUS	Constant+	50.87	2.35	
	BAIT_LR*	2.39	1.11	.20
OCI-R_Checking	Constant	.57	.51	
	BAIT_HR*	.44	.20	.21
PPI_Total	Constant+	113.78	3.60	
	BART_Explo**	.91	.33	.30
PPI_Factor1	Constant	−.02	1.11	
	BAIT_Ins*	−.40	.17	−.25
	BART_Explo*	.15	.07	.23

Abbreviations same as in Table 1. For all regressions, BAIT_Ins, BAIT_LR, BAIT_HR, BART_PAdjAvg, BART_Explo, and BART_$Total were entered as predictors. Only listing coefficients for significant BART or BAIT predictors (p<.05). Regressions only included participants who had a positive trial correlation between insurance purchased and number of required pumps.

*p<.05,

**p<.01,

+ p<.001.

Since more than 5% of participants were missing complete data on the IUS, it was possible that observed relationships with the IUS were dependent upon the values we imputed for missing responses on the questionnaire. To investigate whether this was the case, we redid all analyses with the IUS on the subset of participants who had complete IUS data. The IUS remained significantly correlated with BAIT_LR among subjects who were sensitive to the number of required pumps, and indeed was now significantly associated with BAIT_LR in the larger sample of subjects (including those who were not sensitive to the number of pumps (Kendall's Tau = .14, p<.05)). IUS scores were now also positively correlated with BAIT_Ins among individuals sensitive to the number of required pumps (Kendall's Tau = .15, p<.05). These results indicate that our observed associations with the IUS were not an artifact of our imputation of missing values, and suggest an even stronger relationship between BAIT performance and the IUS than do our data that includes the imputed values.

As a supplemental analysis, we investigated whether the self-report scores that were significantly correlated with the aggregate amounts of insurance purchased were also associated with the amount of insurance purchased at the individual trial level. To do so, we used Generalized Estimating equations (GEE) which allows one to model effects while accounting for correlations within observations of individual subjects [19]. Using PASW Statistics 18 (SPSS Inc., Chicago, IL), we created GEE models with an exchangeable correlation matrix and a normal distribution to predict the amount of insurance purchased on each trial of the BAIT. For consistency these analyses were restricted to subjects who showed a positive correlation between the amount of insurance purchased and the number of required pumps on BAIT trials. Separate models were created to examine the relationship between each self-report score and BAIT performance. For all models, independent variables included the total amount in the permanent bank at the beginning of the trial (i.e. net profit from all prior trials on the BAIT and BART) and the required number of pumps for that trial of the BAIT. Additionally, each model contained a third independent variable – scores on one of the self-report scales. Including trial level variables allowed us to see whether the amount of insurance purchased was associated with individual difference measures after taking into account the risk and the amount of potential loss (i.e. the risk of loss increases as the required number of pumps increases and the amount of potential loss increases as the total amount in the permanent bank increases).

The total amount of money in the permanent bank and number of required pumps were both positively associated with the amount of insurance purchased in each GEE model (all β>.25, p<.001). This was expected because individuals should buy more insurance both when the amount of potential loss is greater and as the riskiness of the unlucky balloon popping increases. Consistent with the primary analyses, the total score on the DOSPERT and the PPI factor 1 score each significantly predicted the amount of insurance purchased (each significant at p<.05; β = −.13 and −.17 respectively). All other self-report scales, with the exception of the OCI-R checking score, predicted the amount of insurance purchased at trend level (p<.10). Notably, the direction of the relationship between each scale and amount of insurance purchased on each trial was the same as that of any significant correlations with that scale and overall measures on the BAIT (i.e. BAIT_Ins, BAIT_InsLR, or BAIT_InsHR). To further see if scores on the OCI-R checking scale were related to BAIT behavior, we performed an identical GEE to that performed before, except this time it was used to only predict behavior on high risk BAIT trials. We limited our prediction model to these trials because the significant positive correlation between overall BAIT performance and OCI-R checking was only significant for high risk trials (i.e. BAIT_InsHR). This GEE did in fact reveal that scores on the OCI-R checking scale were significantly positively correlated with the amount of insurance purchased on each trial (p<.05, β = .11).

## Discussion

We have introduced the BAIT as a potential tool for use in studying individual differences in protective risk management tendencies that are not captured by existing objective risk assessment tasks. Differences in protective risk management tendencies influence a wide range of decisions, ranging from financial investments to proactive safety actions. Moreover, excessive or deficient protective risk management decisions may play a role in certain forms of psychopathology. For instance, obsessive-compulsive disorder may be viewed as reflecting excessive risk management, while individuals with psychopathic personalities may show a failure to manage potential risks. Given the broad range of situations that are influenced by protective risk management, we believe there is a significant need for objective techniques for measurement of these individual differences. The BAIT, which assesses how much one is willing to pay to protect what one already has, was designed to fill this need.

As a preliminary step in validating the BAIT, we found that it shows correlations with self-reported risk attitudes and personality traits that are logically related to risk management. For instance, the amount of insurance purchased was associated with less positive attitudes towards risk as measured by the DOSPERT (both Total and Health/Safety subscale scores), and greater intolerance of uncertainty as indexed by the IUS. In the personality domain, the amount of insurance purchased was associated with greater checking symptoms on the OCI-R and fewer psychopathic personality traits as measured by the PPI Total and PPI Factor 1 scores. With the exception of the PPI Total Score, the BAIT significantly predicted the self-report data, even after entering BART performance into the equations, providing evidence of incremental validity.

Further supporting the potential utility of the BAIT procedure, the amount of insurance purchased on the BAIT outperformed the BART in predicting attitudes towards uncertainty and risk, and in predicting obsessive compulsive symptoms. These relationships provide support that the BAIT captures a distinct construct which may be associated with protective behavior not captured by other risk taking tasks. One strength of the BAIT is that it resembles real situations in which individuals spend money or perform behaviors to protect themselves from harm or loss and to limit risk, and may perhaps capture such behavior better than self report scales that may be influenced by report biases such as socially desirable responding.

The present study provides only an initial test of the BAIT. Future studies of its psychometric properties, including its test-retest reliability and its ability to predict real-world risk management behaviors are clearly needed. An important caveat also must be noted in that not all individuals showed performance that tracked with the apparent risk level on a given trial. Fourteen percent of the subjects failed to buy more insurance on trials that required more pumps. We considered this a validity check, and based our primary analysis only on the 86% of subjects that showed this sensitivity to risk. Consistent with this approach, many of the observed correlations with personality and self-reported risk management attitudes only emerged when the participants insensitive to this index of risk were excluded.

However, it remains unclear why some of the participants failed to show a normal modulation of behavior based on the number of required pumps. These participants may have not properly understood the task and/or may have utilized different performance strategies. Since individuals are told that the unlucky balloon only is revealed if it pops, some may have believed it occurred on an early trial. Other individuals may have primarily attended to trial number, assuming that since the unlucky balloon hadn't yet occurred it was more likely to occur on later trials. Given that individuals accrue more money throughout the task, the risk of monetary loss and also the benefit of buying insurance increases as the task progresses. The majority of participants were clearly sensitive to this, buying more insurance as trial number increased, but it is possible that some subjects followed this strategy to the exclusion of other factors. It is also possible that some individuals may fail to modulate their behavior by the number of required pumps due to a pathological level of excessive or deficient risk management bias. One could imagine for instance an individual with obsessive compulsive disorder always purchasing insurance despite its high cost. Conversely, an individual who is thrill seeking might enjoy the gamble of taking the risk on trials with a high number of required pumps (especially given that the amount of money involved is relatively small). Consistent with this possibility, a few subjects appeared to buy less insurance on trials with a higher number of pumps. To better understand the source of individual differences in BAIT performance it would be beneficial to include a debriefing in which individuals are asked about their strategy on the task.

An interesting finding in the present study is the extent to which the BAIT showed correlations with specific features of personality and risk attitudes. The amount of insurance purchased on low risk trials of the BAIT was positively associated with intolerance of uncertainty. This was predicted a priori as the BAIT by design involves uncertainty. Participants do not know which trial on the BAIT contains an unlucky balloon or whether it will pop. Buying more insurance on the BAIT helps individuals reduce their chances of an uncertain monetary loss. In contrast, BAIT performance was not significantly correlated with the PSWQ, which measures worrying, but is not specific to situations with uncertainty. Indeed, prior research has shown that while scores on the IUS are associated with behavior on tasks with moderate levels of ambiguity, scores on the PSWQ are not [20]. We also found that behavior on the BAIT was not associated with the tendency to tolerate and avoid discomfort as measured by total scores on the DIS, a scale which has previously been associated with fear reactivity to a stressor [12]. This suggests that behavior on the BAIT is related specifically to intolerance of uncertain situations, rather than with intolerance of uncomfortable physiological reactions that can occur in such situations.

The DOSPERT measures risk attitudes by asking individuals how likely they would be to engage in various risky activities. Individuals who report they are less likely to engage in such activities would be expected to buy more insurance on the BAIT, since buying more insurance reduces the risk of the task. Indeed this is what we found for the DOSPERT total score, which suggests that BAIT performance may be associated with risk attitudes across different content domains. However, BAIT performance was most clearly associated with scores on the Health/Safety subscale, as opposed to the financial subscale, which did not reach statistical significance. This may seem surprising given that the BAIT entails monetary rather than health risks. However, this may partially reflect the participant sample, which involved college students, most of whom have only limited experience with independently managing budgets or investments.

As individuals reported greater levels of checking symptoms on the OCI-R, the amount of insurance bought on high risk trials of the BAIT increased. This relationship was robust, as it was also significant across the entire group of participants, including those that did not modulate their behavior based on the number of pumps required on each trial. This association may be partially related to an intolerance of uncertainty in individuals with obsessive-compulsive traits, as the IUS and OCI-R checking scale were themselves significantly correlated (Kendall's Tau = .34, p<.001).

The number of reported psychopathic personality traits was negatively associated with how much insurance individuals purchased on the BAIT. Psychopathic personality traits were also associated with performance on the BART: as psychopathic personality traits increased, the number of balloons pumped up until they popped on the BART increased, which is consistent with prior research [4]. However, both the BAIT and the BART were independently associated with scores on the PPI. After taking into account BART behavior, there was still a significant negative relationship between amount of insurance purchased on the BAIT and scores on PPI Factor 1. This particular factor of psychopathy is associated with the emotional traits of primary psychopathy such as fearlessness [18], [21]. Individuals with high scores on PPI factor 1 may have low fear of punishment on the BAIT and consequently buy little insurance.

Relationships between performance on the BAIT and PPI factor 1 scores and DOSPERT total scores were particularly strong. Scores on both of these scales were negatively correlated with the average amount of insurance purchased and also were predictive of the amount of insurance purchased at the individual trial level after taking into account the amount of money in the permanent bank at the beginning of the trial and the required number of pumps on that trial. The other scales that showed relationships with aggregate BAIT performance predicted individual trial performance at the trend level, with the exception of the OCI-R checking scale, which significantly predicted BAIT trial performance on BAIT high risk trials. In considering these trend level findings, it is important to note a potential confound in these analyses that may have reduced our ability to predict decisions based on trait measures. Specifically, the total amount in the permanent bank is dependent on both performance on the BART and on the amount of insurance purchased on prior trials of the BAIT. Self-report variables that predict BAIT performance are likely to not only be associated with the amount of insurance purchased on the current trial but also that purchased on previous trials (i.e. amount of money in the permanent bank). Similarly, any personality traits predicting BART performance will contribute to the total amount of money in the permanent bank on the BAIT (especially on earlier trials). To avoid an influence of the total amount of money won on the BART, we suggest that investigators endow subjects with a little extra money, in order to have all participants start the BAIT with the same amount of money in their permanent bank. In contrast, the fact that previously purchased insurance impacts the amount of money in the permanent bank cannot be easily corrected for while maintaining the ecological validity of the task. Because of this, we believe that probing for relationships at the aggregate level, as was done in our primary correlation and regression analyses, provides the most power for detecting differences in risk management traits.

In summary, the present data provide support for the potential utility of the BAIT as an index of risk management biases that compliments the BART. We found that the BAIT showed associations with personality and risk attitude measures after controlling for the BART and that many of these measures were not correlated with the BART, indicating that the BAIT captures unique individual differences in risk management style. In order to facilitate its inclusion in future research studies, the BAIT will be made available for download upon request to the senior author, or by download at http://www.addiction.umd.edu/downloads.htm.

## Supporting Information

Table S1 Correlations with the BAIT for Participants with a positive trial correlation between insurance purchased and number of required pumps.
(DOC)
