# Supplementary material for: The Balloon Analog Insurance Task (BAIT): A Behavioral Measure of Protective Risk Management
Source: PLoS One. 2011 Jun 28;6(6):e21448. doi: 10.1371/journal.pone.0021448 (PMC3125190; doi:10.1371/journal.pone.0021448)
Supplement: Table S1 — Correlations with the BAIT for Participants with a positive trial correlation between insurance purchased and number of required pumps. (DOC) [file pone.0021448.s001.doc]

| Variable | Mean | SD | BAIT_Ins | BAIT_LR^nn^ | BAIT_HR |
| --- | --- | --- | --- | --- | --- |
| BAIT_Ins | 4.32 | 1.84 | - | .80+ | .92+ |
| BAIT_InsLR^nn^ | 1.79 | 1.12 | .80+ | - | .56+ |
| BAIT_InsHR | 2.49 | .85 | .92+ | .56+ | - |
| BART_PAdjAvg | 40.25 | 15.40 | -.06 | -.01 | -.03 |
| BART_Explo^nn^ | 10.55 | 4.15 | -.05 | -.03 | -.02 |
| BART_$Total^nn^ | 7.24 | 1.74 | .01 | .03 | .04 |
| Age^nn^ | 20.22 | 1.68 | -.05 | -.06 | .04 |
| Gender (% female) ^nn^ | .55 | .50 | -.07 | -.08 | -.10 |
| DIS^nn^ | 11.65 | 5.31 | .03 | .06 | .02 |
| DOS_Total | 2.53 | .42 | -.19* | -.09 | -.15 |
| DOS_Ethical^nn^ | 1.96 | .62 | -.06 | -.04 | -.06 |
| DOS_Financial^nn^ | 2.11 | .55 | -.06 | -.06 | -.04 |
| DOS_Health/Safety | 2.25 | .62 | -.21* | -.14* | -.17 |
| DOS_Recreational | 3.11 | .76 | -.12 | -.08 | -.11 |
| DOS_Social^nn^ | 3.24 | .56 | .02 | .01 | .06 |
| IUS^nn^ | 55.15 | 13.30 | .12 | .14* | .10 |
| OCI-R_Total^nn^ | 12.88 | 9.15 | .05 | .07 | .08 |
| OCI-R_Checking^nn^ | 1.65 | 1.77 | .10 | .09 | .14* |
| OCI-R_Hoarding^nn^ | 3.15 | 2.74 | .02 | .04 | .06 |
| OCI-R_Neutraling^nn^ | 1.17 | 1.71 | .05 | .07 | .07 |
| OCI-R_Obsessing^nn^ | 1.77 | 2.08 | .11 | .13 | .04 |
| OCI-R_Ordering^nn^ | 3.46 | 3.13 | -.04 | -.02 | .04 |
| OCI-R_Washing^nn^ | 1.54 | 2.18 | .02 | .05 | .02 |
| PPI_Total | 122.86 | 13.40 | -.23* | -.17* | -.19 |
| PPI_Factor1 | -.18 | 2.90 | -.28* | -.13 | -.27* |
| PPI_Factor2 | -.10 | 2.03 | -.02 | -.06 | .04 |
| PSWQ | 41.76 | 11.55 | .12 | .06 | .16 |

Abbreviations for BART and BAIT variables are the same as in Table 1. DIS: DIS Total Score. PSWQ: PSWQ Total Score. OCI-R_Total: OCI-R Total Score. Other variables beginning OCI-R are total scores on that subscale. IUS: IUS Total Score. DOS_Total: DOSPERT Total Score. All other variables beginning DOS are total scores on that subscale of DOSPERT. PPI_Total: PPI Total Score. PPI_Factor1 and PPI_Factor2: total scores on factors 1 and 2 of PPI (details of how these factors created in text). nn: variable is significantly non-normal according to Kolmogorov-Smirnov test, and all correlations with this variable are values of Kendall’s Tau. All other correlations are Pearson correlations. N = 110 for all correlations except for correlations with Age (N = 108), OCI-R_Neutralizing (N = 109), OCI-R_Washing (N =109), and PPI variables (N = 78). * p < .05 (2-tailed), + p < .001 (2-tailed).
